# “Meet us where we’re at:” Towards engaging and inclusive research with young adults with a lived experience of cancer

**DOI:** 10.1017/cts.2025.10101

**Published:** 2025-07-16

**Authors:** Nicole Collaço, Céline Bolliger, Kirsten Efremov, Peter Dawes, Sonia Obiokafor, Anne-Sophie Darlington, Samantha Sodergren

**Affiliations:** 1 School of Health Sciences, University of Southampton, Southampton, England; 2 Faculty of Health Sciences and Medicine, University of Lucerne, Lucerne, Switzerland; 3 Swiss School of Public Health, Zurich, Switzerland; 4 Patient Partner, Ontario, Canada; 5 Patient Partner, Southampton, England

**Keywords:** Cancer research, young adults, research participation, inclusivity, diversity

## Abstract

**Background/Objective:**

Meaningful engagement with young adults (YAs) with a lived experience of cancer is important for conducting impactful research on issues that matter to them, and ensures their voices are central to shaping cancer research outcomes. This preliminary study explored barriers and facilitators to participation in research to identify strategies for making cancer research more inclusive and responsive to the needs of YAs.

**Methods:**

This qualitative study involved twelve YAs (aged 21–43 years at time of interview) with a lived experience of cancer, who participated in a focus group or interview. Participants were recruited via multiple cancer charities/organizations and social media platforms. Data were analyzed using thematic analysis.

**Results:**

Barriers to research participation were Person Specific (*health and wellbeing, logistical and practical challenges, knowledge, understanding and confidence*) and Systemic (*lack of advocacy, social and cultural influences*). A multi-pronged approach to engage YAs in cancer research should include framing research to make it more relatable, using accessible language, and showcasing its potential value and impact. Incentivising participation and offering flexible engagement formats, (e.g., online surveys and videos), to *meet individuals where they are,* can aid participation. Collaboration with trusted organizations, ensuring diverse representation in recruitment materials, and using social media platforms were recognized as effective ways to reach a broader audience and ensure inclusivity.

**Conclusions:**

We provide practical strategies on how to implement these approaches. From a researcher perspective, early consideration of funding allocation (e.g., dedicated person for social media engagement, time of Patient and Public Involvement) is key to support these strategies and enhance engagement.

## Introduction

A cancer diagnosis is challenging at any age, but for adolescents and young adults (AYAs), defined as those aged 15–39 years at diagnosis [1]; cancer disrupts significant milestones/life transitions. These include building a career, gaining (financial) independence, forming relationships, starting a family, and developing a sense of self, including sexual and personal identity [2,3]. AYAs also face distinct biological and systemic obstacles, including more aggressive cancers [4,5], delays in diagnosis due to low cancer suspicion, limited clinical trial access, care inequalities, poorer treatment adherence, and lack of policy attention [6].

To improve outcomes for AYAs with cancer, it is essential to better understand and address their needs and concerns during and beyond treatment through research, which can, in turn, inform age-appropriate healthcare and support. “Health research” refers to research aimed at improving health outcomes, experiences, and services which extends beyond clinical trials to include qualitative and mixed methods studies. While AYAs have long been acknowledged as playing an important role as participants in health research, their potential as active research contributors and collaborators has also been recognized to optimize research quality in terms of design, delivery and dissemination [7,8].

Despite the acknowledgment of the importance of their involvement, AYAs remain under involved in health research [9]. In particular, the issue of diversity and achieving meaningful representation of individuals from minority groups (ethnicity, sexual orientation and gender identity, disability, socio-economic status) remain challenging [10,11]. Achieving greater diversity in health research is important to understand and address health inequities [12,13]. It is acknowledged that such individuals are seldom included in research due to the exclusionary models of research design and delivery [14]. Other common barriers include lack of awareness about research opportunities, difficult-to-understand terminology used to explain studies and clinical trials, lack of trust in how their data will be used, largely due to poor communication, structural issues such as parental consent requirements and financial instability [10,15,16]. Researchers also face challenges developing sustainable, trusting relationships, particularly with underserved communities, where building such connection requires considerable time and resources [17].

A growing body of literature provides suggestions for improving AYA participation in cancer research. These include better communication about the trial/study, flexible research methods, streamlined ethical processes, education of clinicians to better communicate trials to AYAs, promoting diversity in research team composition, using social media, building trust and ensuring that AYAs feel supported, listened to and valued [10,13–15,18]. Whilst these strategies outline what is important, there is limited guidance on *how* researchers can practically implement them at different stages of the research process. This gap in actionable guidance leaves researchers unsupported to do so.

This current preliminary, exploratory study builds on existing evidence and aims to further explore the barriers to engaging AYAs with lived experience of cancer in research, particularly those from underrepresented backgrounds, and provides recommendations on how best to improve recruitment of AYAs in cancer research. Unlike previous studies, which have primarily focused on barriers to participation in clinical trials or provided broad, high-level recommendations, our research aims to explore engagement across a wider range of research contexts. Importantly, we will offer practical guidance on how researchers can improve inclusivity throughout the research process. Co-designed with the target population, these solutions can serve as a foundation that researchers can build upon to inform more impactful research practices that genuinely reflect their needs and priorities.

## Methods

The Consolidated Criteria for Reporting Qualitative Research (COREQ) guidelines [19] were followed in reporting the study findings (Supplementary file 1).

### Study design

This exploratory qualitative study employed a focus group methodology to foster interactive discussions and elicit diverse perspectives on shared experiences [20]. This study was conducted between January and August 2024. Two young people with lived experience of cancer (KE, PD) formed part of the research team and provided strategic advice, including input on the topic guide and patient information materials.

### Participants and recruitment

Inclusion criteria for this study were AYAs aged 16 and 39 years old at the time of their initial cancer diagnosis. We aimed to recruit up to 15 participants, attempting for varied representation from varied backgrounds (e.g., ethnicity, disability, sexual orientation, socioeconomic status), as well as age and sex. The inclusion criteria were deliberately broad to ensure accessibility to a wide range of participants. Although we sought diversity, we did not adopt a fixed quota for specific demographics, our objective was to be inclusive in our recruitment strategy with the only inclusion criteria being age range and a diagnosis of cancer. Recruitment utilized a combination of purposive, snowball and convenience sampling and was conducted through a multi-pronged approach aimed at reaching young people across various settings:

To reach a broad and diverse group of participants, we utilized multiple platforms and contacted 18 cancer charities/foundations supporting young people with cancer, covering different cancer types as well as those specifically supporting people from underserved groups. We collaborated with Shine Cancer Support, Black Women Rising and the Surrey Minority Ethnic Forum to disseminate study information through their existing networks, including multiple social media platforms; their closed Facebook groups, newsletters, and WhatsApp groups. Study information was also shared on the research group’s social media platform “X,” using relevant hashtags. Maggie’s Centre in University Hospital Southampton also displayed posters in their facility. Recruitment posters were displayed in locations frequented by young people, including university campus, cafes, and community centers.

Interested participants were invited to contact the researchers and were sent an information sheet. Researchers (NC and SS) conducted a short introductory call with each participant providing an opportunity to get to know participants, discuss the background to the research and answer any questions before deciding to give their consent to participate.

### Ethical considerations

Ethical approval for this study was obtained from the University of Southampton (ERGO 90913). All participants provided informed consent prior to participation through recorded video consent. To ensure confidentiality, all data were anonymized, and participants were assigned unique identification numbers.

### Data collection

Four focus groups were conducted with group sizes ranging from 3 to 5 participants: one in person and three online using Microsoft Teams. One online interview was conducted with a participant who was unable to attend the focus groups. Focus groups were facilitated by NC and SS who are trained female researchers with extensive experience in qualitative methods and working with young people with cancer. They had no prior relationship with the study participants but have an interest in supporting young people with cancer. A semi-structured interview guide was developed and reviewed by the research panel, of which two members have a lived experience of cancer. The interview guide (Supplementary file 2), covered topics such as: personal experiences of barriers to engagement in research, and what was missing from current research practices for inclusive research. For many, this was their first research experience, ensuring perspectives beyond those frequently involved/familiar in/with research. Focus groups lasted approximately two hours and were video-recorded using Microsoft Teams with participant consent. Field notes were written during the focus groups/interview to aid the analysis process and supplement the Microsoft Teams transcription. NC verified transcripts for accuracy. Participants were asked to complete an online demographics form.

### Data analysis

**Figure 1.**
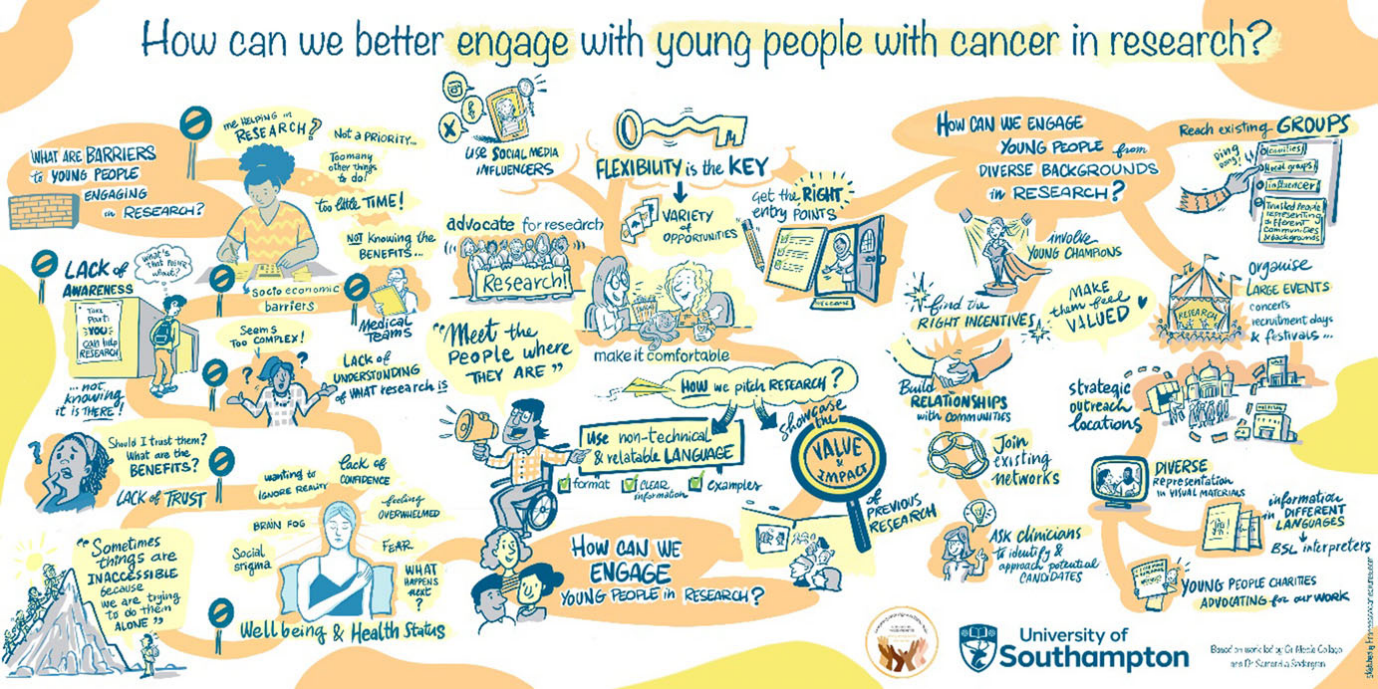
Digital illustration representing the key themes and insights emerging from the focus group discussions on how we can better engage young adults (YAs) with cancer in research.

Video recordings were transcribed verbatim. Data were analyzed using thematic analysis [21], following a six-step process of familiarization, code generation, theme development, theme review, theme definition, and report writing. Initial codes were generated, and coded data were read independently by the researchers (NC and SS). Data were then grouped into higher order themes, by identifying patterns, synthesizing codes that overlapped and separating out distinct codes. The coded data were collated and organized into themes through research team discussions, allowing refinement and review. Microsoft Word and Excel were used to manage the data. To enhance the accessibility and impact of the findings, a digital illustrator was commissioned to create visuals representing the key themes and insights emerging from the focus group discussions, working closely with the lead researchers to accurately reflect the experiences of the participants (See Figure 1). While data saturation was not the primary focus of this exploratory study, limitations of time and resources meant that we were guided by the principle of information power [22] to support our decision to close recruitment.

## Results

Twelve participants took part in this study. While seventeen individuals initially expressed interest, one was ineligible due to being under the age of 16 years at cancer diagnosis, and four did not respond after receiving study information. Participant characteristics are detailed in Table 1. In summary, the sample consisted of 11 females and 1 male, aged between 18 and 38 years at time of diagnosis, and 20 and 44 years at time of participation, representing a range of cancer types, treatment statuses (7 on treatment, 5 who had completed treatment) and disability status (*n* = 4). Participants also varied in ethnicity, with the majority being White (66.7%), alongside Black, Caribbean or African (16.7%), Bangladeshi (8.3%) and mixed or multiple ethnic groups (8.3%).

**Table 1.** Characteristics of participants

Characteristics	Number (%)
**Current age groups**	
20-24 years	2 (16.7%)
25-29 years	2 (16.7%)
30-34 years	2 (16.7%)
35-39 years	3 (25%)
40-44 years	3 (25%)
**Age groups when diagnosed with cancer**	
16-19 years	1 (8.3%)
20-24 years	2 (16.7%)
25-29 years	1 (8.3%)
30-34 years	4 (33.3%)
35-39 years	4 (33.3%)
**Treatment**	
On treatment	7 (58.3%)
Off treatment	5 (41.7%)
**Type of cancer**	
Acute lymphoblastic leukaemia	1 (8.3%)
Acute myeloid leukaemia	1 (8.3%)
Essential thrombocythaemia	1 (8.3%)
Myxoid Liposarcoma	1 (8.3%)
Hodgkin’s Lymphoma	2 (16.7%)
Brain tumour	1 (8.3%)
Breast cancer	5 (41.7%)
**Gender**	
Female	11 (91.7)
Male	1 (8.3%)
**Ethnic group**	
White	8 (66.7%)
Black, Caribbean or African	2 (16.7%)
Bangladeshi	1 (8.3%)
Mixed or multiple ethnic groups	1 (8.3%)
**Education**	
Other (diploma)	1 (8.3%)
University	8 (66.7%)
Post-compulsory education below university (e.g., college or vocational qualifications)	1 (8.3%)
Compulsory education completed	1 (8.3%)
Not answered	1 (8.3%)
**Employment status**	
Sick leave	1 (8.3%)
Full-time	5 (41.7%)
Part-time	3 (25%)
None- student	2 (16.7%)
Homemaker	1 (8.3%)
**Disability**	
No	7 (58.3%)
Yes	4 (33.3%)
Not answered	1 (8.3%)
**Disabilities**	
Cancer diagnosis	1 (8.3%)
Adrenal insufficiency	1 (8.3%)
Partially blind	1 (8.3%)
Not answered	1 (8.3%)
**Location**	
South East England	8 (66.7%)
South West England	1 (8.3%)
West Midlands	1 (8.3%)
Scotland	1 (8.3%)
Wales	1 (8.3%)
**Heard about study**	
Maggie’s Centre	1 (8.3%)
FB post through Shine	7 (58.3%)
Black Women Rising Whatsapp/newsletter	3 (25%)
Other	1 (8.3%)

The focus groups/interview explored three key areas: (i) barriers to research participation, (ii) strategies for engagement in research, and (iii) methods for ensuring inclusivity and engagement of individuals from diverse backgrounds. The participants from hereon will be referred to as YAs given their age at participation falling within the young adulthood part of the AYA definition.

**Figure 2.**
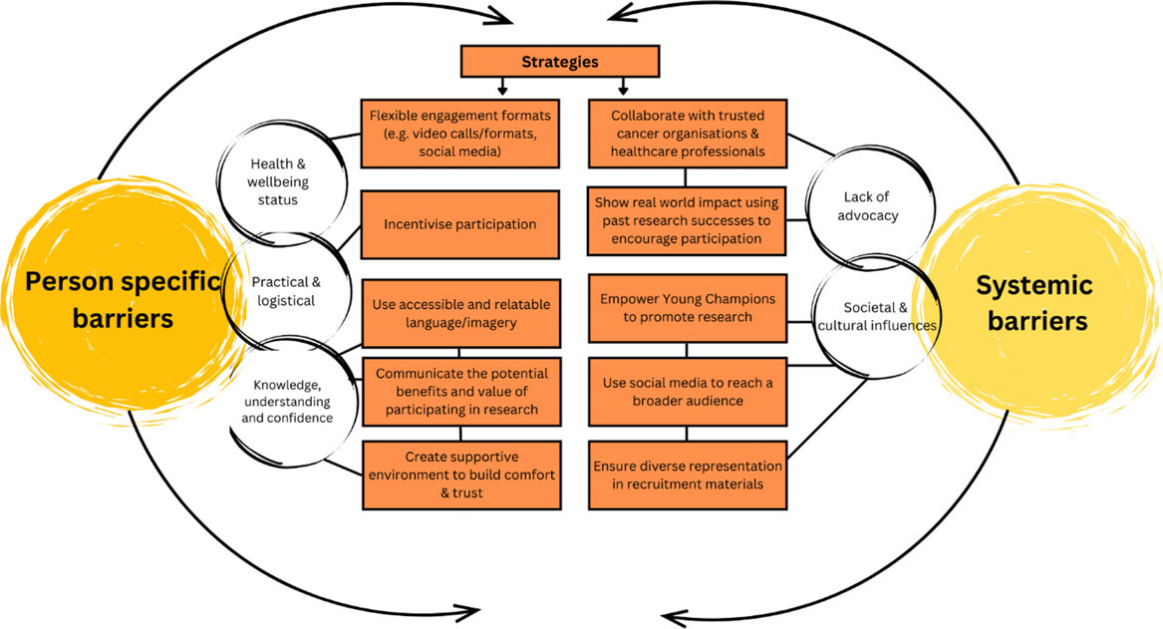
Barriers and strategies to engaging young adults (YAs) with a lived experience of cancer in research.

Please see Figure 2 for barriers and strategies to engaging YAs in research.

***What are the barriers to engaging young adults in research?***

### Person specific barriers

Person specific barriers related to individual health, emotional state, and personal circumstances that hindered participation in research.

### Health and well-being status

Health and well-being of individuals were an important factor for YAs considering research participation. Managing cancer treatment and its side effects could make participation in research a lower priority: “*you’re just so busy with all of the treatment and the appointments…obviously you need to prioritize that […] the medication makes you too weak or tired all the time.”* [FG4PM01-female, diagnosis age 38, participating age 43]. The emotional toll of having/had cancer also posed barriers, with some feeling it’s an unwelcome reminder of their cancer experience: *“I kind of slowly joined one early stage breast cancer group…that took me like a good six months to kind of actually join those because it’s just too much”* [FG2P2-female, diagnosis age 20, participating age 21].

### Practical and logistical challenges

The demands and responsibilities placed upon YAs coupled with their current health status was also recognized as creating practical and logistical issues which made research participation problematic.

Time constraints, particularly balancing treatment with work, studies or family care could limit availability for research participation:“*[…] the barrier would have been time […] if you’re working, if you’ve got kids…if you’re living with parents…*” [FG104-female, diagnosis age 37, participating age 38]. Financial concerns, especially for those balancing illness and work also deterred participation: *“participating in research… it’s a non-essential thing… if you’re off sick, and you’re only entitled to a certain amount of full sick pay… if you’re expected to travel to attend something [research activity] in person, that might mean that you’re losing out on a day’s pay, …any incentive you might be able to give might not make up for that”* [FG4PM01-female, diagnosis age 38, participating age 43]. Geographic and socioeconomic disparities further exacerbate accessibility issues: *“[…] we don’t really get opportunities like this unless we see it on social media. Your financial status might not make it possible to be involved as well*” [FG2P2-female, diagnosis age 20, participating age 21].

### Knowledge, understanding and confidence

A lack of awareness and misconceptions about research, it’s importance and the process may deter engagement: *“A lot of it is you have to advocate for yourself… if people don’t know that there is such things as studies and research”* [FG101-female, diagnosis age 39, participating age 44]. A lack of foundational knowledge of what research is could lead to misconceptions and apprehension. Participants voiced concerns such as, *“the word research just screams papers and academic”* [FG4PM02-male, diagnosis age 18, participating age 28] and, *“Depending on your background… people wouldn’t understand what research is apart from my peers that have been to university… Do they know how they can engage with it? Is it to scientific for me? Is it too intelligent for me? Is it a place where I belong?”* [FG103-female, diagnosis age 33, participating age 33].

Some lacked the confidence to contribute to research: “*I wouldn’t have felt confident to go into a research setting and kind of like share/ think that my opinions are kind of worth listening to*…*”* [FG104-female, diagnosis age 37, participating age 38]. Additionally, a lack of clarity regarding the benefits and purpose of research could hinder involvement: *“There’s no guarantee of any concrete change as a result of what we say… I do not know how much visibility that people always have in studies about kind of what actually practically changes after”* [FG4PM03-female, diagnosis age 26, participating age 28].

### Systemic barriers

Systemic barriers related to the healthcare and research systems, as well as family and societal influences.

### Lack of advocacy

Inconsistencies in healthcare professionals’ support for research complicated research engagement: *“I hear vast differences… between different specialists as to whether they actually promote the research to the patients or not”* [FG101-female, diagnosis age 39, participating age 44]. This uncertainty was compounded by past experiences of being unheard during their cancer journey: *“… It’s not going to change […] if they weren’t listened to through their cancer journey, what’s going to be the difference now in research?”* [FG3AM01-female, diagnosis age 36, participating age 37].

### Social and cultural influences

In some cases, participants perceived limited encouragement to engage in research in their healthcare journey. Parental protective instincts also acted as a barrier to participation. This challenge could be magnified by a lack of understanding about the research process within some communities: *“[…] Coming from a very, very much working class…would you feel comfortable in a group of your friends saying oh I’m going to get involved in some research?… Is that gonna be accepted? It’s not really the norm, well not seen as normal in certain communities… it’s really difficult to challenge social norms… it’s very easy to kind of just follow the crowd”* [FG103-female, diagnosis age 33, participating age 33]. Perceptions of research as overly burdensome, elitist, or irrelevant to their lives could deter potential participants.

Social stigma around young people with cancer could discourage participation: *“There is a social stigma around having cancer as a young person…and people lose a lot of friends… people do not want to talk about it because a lot of people just want to get that chapter of their lives over and done with”* [FG3AM01-female, diagnosis age 36, participating age 37].

***How can we engage young people in research?***

### Reframing research: making it relatable and approachable

#### Accessible language/humanizing research

Participants emphasized the importance of using simple, relatable language and imagery to make it more approachable. As one participant stated, *“Changing it from calling it “research” to “knowledge bringing, experience gathering”… people see ‘research’ and think, oh, they’re going to be in a lab coat and I’m going to get poked and prodded. it is very much about the language”* [FG3AM01-female, diagnosis age 36, participating age 37].

#### Communicating value and potential impact/benefit

Participants highlighted the need to understand the personal relevance of research. Framing the benefits of taking part in ways that resonate with long term outcomes such as helping others in the future, and also immediate needs, such as how participants can benefit now: *“highlighting the benefits…say “your child might meet some like-minded people, it may help them come to terms with their situation, give them some purpose.” So that kind of changing it instead of “oh, your child’s Guinea pig.” Like this will benefit your child. Rather than, this will benefit us”* [FG2P1-female, diagnosis age 23, participating age 23].

In addition, sharing previous research results and demonstrating impact through relatable narratives and lived experiences can be particularly powerful. Participants recommended incorporating *“quotes from people or like real people or even like pictures and videos”* to illustrate the personal side of research and create a sense of connection [FG3AM02-female, diagnosis age 33, participating age 36]. Sharing stories of how research has led to tangible changes, such as the development of support programs or resources for specific cancer types, can increase engagement in research.

#### Incentivising participation

The use of personalized, meaningful incentives was reported as important. While some participants might be motivated by the opportunity to contribute to meaningful research, others may respond more positively to tangible rewards such as vouchers or giveaways: *“Some people are motivated by freebies or things like Amazon vouchers; those things may well help motivate some people”* [FG4PM01-female, diagnosis age 38, participating age 43]. To avoid incentives that could lead to tokenistic engagement, participants reinforced that the focus should be on fostering meaningful involvement and ensuring young people feel valued for their contribution beyond any rewards.

### Flexible engagement formats

#### Meet people where they are

Participants reported the importance of offering flexible formats to suit diverse preferences, such as online surveys, video calls/formats and social media. Flexibility in location and format acknowledges that in person participation may not always be feasible or preferable. Virtual environments like the metaverse could also provide a comfortable space for those apprehensive about traditional research settings. Researchers should meet participants where they are: *“tap into where [people] are engaged and then bring the research to them,” integrating opportunities within existing platforms (e.g. social media, TikTok and Instagram) and activities favored by young people”* [FG103-female, diagnosis age 33, participating age 33]. This may involve meeting participants within their existing routines and environments, such as offering research participation opportunities within hospitals or treatment centers. Incorporating research into user friendly apps, gamification or providing supportive elements like snacks can make participation more accessible and engaging.

#### Creating a supportive environment: building comfort and trust

A comfortable and supportive environment is essential for fostering positive participation experiences. This includes providing clear introductory information, and easing participants into the research process, through for example, an introductory call: *“I think definitely I would have felt a lot more nervous today if we haven’t had that introductory call. Yeah, so some kind of like instructive video at the very least. Yeah, just so you can see a face.”* [FG2P1-female, diagnosis age 23, participating age 23].

### Engaging through trusted channels and influencers: Right entry points

#### Collaborating with trusted organizations

Participants noted key entry points to engage YAs with a lived experience of cancer in research, including using trusted sources and platforms and collaborating with trusted organizations already embedded within the AYA community. Charities like Shine and Black Women’s Rising were viewed as particularly key, offering a pre-existing level of credibility and trust that could encourage research participation: *“Anytime I’m going to see anything research related to cancer, I always find it helpful to see it on trusted groups… If I see it there, then it’s more likely that I’m going to take part”* [FG4PM02-male, diagnosis age 18, participating age 28].

#### Healthcare professionals

Healthcare professionals, particularly doctors and specialist nurses, were identified as essential for research engagement. Information delivered directly from these trusted people carried significant weight: *“Hearing it from my doctor… I would just do whatever they told me… If they’re saying to me, “we’ve got this research project that might be helpful,” I would have 100% signed up”* [FG102-female, diagnosis age 34, participating age 42]. The existing rapport and trust within these relationships provide a natural avenue for introducing research opportunities in a sensitive and approachable manner.

#### Empowering “Young Champions” to promote research

The concept of “Young Champions” was identified as an effective strategy for promoting research participation. YAs are more likely to get involved in research when they hear about it from other YAs who have had similar experiences. Participants viewed this as a powerful way to connect authentically and build trust: *“If you want to get young people involved, use young people to champion it… you know, people can relate to and they see people or say they we see people that look like us and represent us… that would be championing and raising awareness of it [research]”* [FG103-female, diagnosis age 33, participating age 33].

#### Social media platforms

Participants highlighted the significant role of social media, particularly Instagram, in connecting with and informing individuals about cancer.” Cancer influencers” who share their personal journeys online was identified as a powerful tool for both information dissemination and emotional support: *“Finding someone who’s walking a similar path to you is so beneficial”* [FG3AM02-female, diagnosis age 33, participating age 36].

Furthermore, participants reported the effectiveness of visual communication across social media platforms. Videos, in particular were viewed to offer versatility for dissemination and can resonate with diverse audiences, including those for whom English is not their first language [FG3AM01-female, diagnosis age 36, participating age 37]. The idea of collaborating with young social media influencers, particularly those with cancer-related experiences, could be approached to reach the target audience more effectively.

***How can we engage young people from diverse backgrounds or make research inclusive?***

### Creating partnerships for inclusive research

To effectively engage YAs with lived experience of cancer in research, participants highlighted the importance of building relationships with trusted cancer charities/organizations: “these *groups already have trust and access within these communities,”* making them *“valuable partners”* who can facilitate outreach [FG4PM01-female, diagnosis age 38, participating age 43]. Placing messaging about research studies in familiar spaces frequented by young people and collaborating with community leaders who represented diversity in ethnic and minority groups were viewed as effective ways to reach a broader audience. For instance, one participant suggested that *“translating our stuff and leaving it with the local community leaders is really helpful”* [FG3AM01-female, diagnosis age 36, participating age 37]. Showcasing past successes and the value of research was seen as essential for securing buy-in from potential partners. Successful engagement with existing networks that engage with YAs impacted by cancer was recognized as beneficial; *“Partner with people already doing this work… there’s strength in numbers”* [FG4PM01-female, diagnosis age 38, participating age 43]. This collaborative approach was viewed as essential: *“sometimes things are inaccessible because we’re trying to do them alone.”* [FG4PM01-female, diagnosis age 38, participating age 43].

### Ensuring diverse representation in recruitment materials and discussions

Participants reported the need for inclusivity throughout the research process. This includes ensuring research materials feature diverse voices and experiences and actively involving individuals from a wide range of backgrounds: *“try and have the people that you are asking to get involved… from as much a diverse background as you can”* [FG3AM02-female, diagnosis age 33, participating age 36]. This emphasis on representation extends to the communication and dissemination of research findings. Another participant highlighted the value of providing information in accessible formats, such as videos, and ensuring materials resonate with the target audience’s preferred learning style: “*I think the visual platform is the best way whether it is video, audio, whether it’s simple infographic, I think it’s a really good way of trying to engage with people. […] I think that demographic are far more interested in visual stimulation than they are reading* [FG3AM01-female, diagnosis age 36, participating age 37]. Furthermore, the use of interpreters, including British Sign Language interpreters, was identified as crucial for ensuring equitable access to research opportunities.

Individuals are more likely to engage when they see themselves reflected in outreach efforts, such as relatable figures who share similar backgrounds and experiences: *“You need someone who looks like you and is similar to you”* [FG3MA02-female, diagnosis age 33, participating age 36]. This idea of relatability goes beyond appearances to include shared experiences and social identities. For example, involving individuals from specific communities, and using their language skills to connect with their peers within their communities/background was seen as particularly powerful: *“[…] If you have someone who speaks your language and is not from the UK, who can talk to their community. Then that is really powerful, and that’s something quite simple.* [FG4PM02- male, diagnosis age 18, participating age 28]. Participants also suggested framing research invitations to highlight the value of individual experiences and perspectives, rather than just focusing on “diverse” groups.

## Discussion

This study builds on the existing research on barriers and facilitators to research participation for AYAs with a lived experience of cancer. We also sought to identify strategies for making cancer research more inclusive and responsive to their diverse needs. Our participants represented a range of experiences and backgrounds, including many who had not been previously exposed to research opportunities. Our findings revealed a range of barriers to their engagement in research, including current health related issues and emotional toll of cancer, practical and logistical constraints (time, finances, location), knowledge, understanding and confidence in and or engaging in research, and systemic issues related to social and cultural stigma and influence of healthcare professionals and parents on participation in research. These findings echo other studies which report on similar barriers to research participation in clinical trials [24–27]. However, our study adds new insights, including the emotional burden of cancer and treatment, which makes participation feel like an unwelcome reminder, as well as the importance of timing of participation which can impact willingness depending on treatment stage and emotional readiness. A lack of confidence in taking part in research was also a significant deterrent. Additionally, social stigma, particularly in certain communities where having cancer as a young person is taboo or research involvement is not considered acceptable/normal, further discourages participation. This barrier is not fully addressed in existing research on AYAs with cancer, although it has been noted in broader literature in adults [28]. The unique developmental and social challenges faced by YAs can amplify the impact of this stigma making it particularly important. Understanding and addressing these barriers requires a multi-faceted approach that meets practical needs, builds trust in the research process, develops relationships with the right people, and ensures equitable access to research opportunities.

In terms of strategies for improving engagement, participants suggested that dispelling myths about research and making it more accessible could help overcome barriers. Using clear, relatable language and pitching research appropriately aligns with previous research [11,29], highlighting the importance of involving young people early in co-producing research activities to ensure relevance, relatability and accessibility. Offering recruitment materials in multiple formats, languages and showcasing diversity of people in recruitment materials has been previously reported to bridge gaps in understanding and engagement, particularly for ethnic minority communities [29]. Participants also emphasized the importance of flexible formats to engage, such as online surveys, utilizing visuals (infographics) and using social media. This recommendation is consistent with the growing body of evidence supporting the use of digital/social media platforms to improve access to research, particularly for younger populations [24,27,30,31]. Furthermore, addressing practical barriers by covering accessibility costs, offering appropriate incentives [24] and designing flexible recruitment strategies showcases respect for young people’s time and circumstances. Despite its significance, the need to show participants the tangible impact of their involvement/how research is framed is not widely discussed in the literature, though two studies have highlighted this gap [32,33]. This suggests that researchers should focus on communicating the potential personal value of participation, such as fostering a sense of purpose or providing opportunities to connect with others facing similar experiences.

Developing relationships with leaders of specific communities and organizations was identified as a key facilitator to engaging young people from diverse backgrounds in research. This approach aligns with existing literature highlighting the importance of early and relationship-focused engagement. Gafari *et al* [29] argue that building connections with communities through face-to-face interactions is important for fostering trust. Whilst participants in our study also recognized this, we found broad approaches to identifying young people with cancer in the community proved less effective. In contrast, working with cancer charities trusted by young people was more successful. These organizations provided access to established networks where trust had already been built, ultimately, facilitating recruitment. In certain parts of the United Kingdom, *community engagement officers* act as valuable links, bridging the gap between research and underserved communities. Collaborating with these engagement officers can be instrumental in ensuring that research reaches and resonates with diverse groups of young people, as they have already established key relationships with these organizations and individuals.

A novel insight from our findings is the important role of “young champions” in engaging young people in research, a concept not widely explored in the literature. Training programs for lay health educators are more effective when tailored to specific populations and utilize a” Train the Trainer” model, empowering community members to educate their peers [34,35]. This approach has been shown to improve knowledge of and attitudes towards cancer clinical trials, as well as breast cancer screening and research. For example, a Breast Health Research Champion program successfully trained women to become community advocates, increasing their knowledge and confidence while positively influencing the attitudes and behaviors of individuals in their social networks [36]. Similarly, employing dedicated staff to promote research within clinical settings has been shown to significantly increase the likelihood of patients being approached about research participation [37].

Meaningful inclusion in research goes beyond categorizing individuals or tokenistic representation, although acknowledging disparities is important. Cultural sensitivity in research, including the need for diverse representation among healthcare providers and researchers are needed [38]. As Preston *et al* [39] state, “*Diversity isn’t just about the demographics of those involved but about the variety of approaches taken to ensure individuals are approached and involved in ways that accommodate their needs and lifestyles*.” Individuals may identify with intersecting characteristics/identities, and thus individuals are not always neatly captured by these categories (e.g. disability, socioeconomic status); there is a richness and complexity within these groups that requires a more nuanced approach to inclusion.

Despite available guidance, drawing on research and public involvement literature, on engaging underrepresented groups in research [1,14,17,23,40–44]; practical application remains challenging, and the issue of representativeness and inclusivity persists. This is partly due to barriers researchers face (see Table 2), including navigating ethical guidelines that restrict how researchers communicate research benefits, funds to manage incentives, and institutional requirements (e.g., lengthy consent processes/information sheets, which can be off-putting to young people and safeguarding issues with social media). Effectively engaging young people requires specific skills, resources, and dedicated time, often overlooked in traditional research settings. Providing training to researchers on accessible communication, cultural competency, age-related issues, and ethical engagement practices is important for successful implementation [40,45].

**Table 2.** Barriers and key recommendations/actions to engage young adults (YAs) with a lived experience of cancer in research- a step-by-step checklist

Barriers for researchers	Recommendations
**BUDGETING AND PLANNING A STUDY**	
Securing adequate funding for patient engagement. Short-term grants may not provide the sustained financial support needed for ongoing community engagement and relationship-building beyond the initial research project.	***Budget for research engagement expenses*** Include participant related expenses in budget such as travel, accommodation costs (for in person research activities) and incentives. Consider financial compensation for people and organizations offering access to participants. ***Plan for accessibility*** Budget for translation services, culturally appropriate media, and accessible materials/formats (e.g. braille, audio, and easy-read documents, along with video captions).
Unfamiliarity of researchers of the trusted organizations and support networks that young people with cancer access. Limited resources and competing priorities of researchers and organization impeding collaboration. Coordinating partnerships with multiple organizations and community stakeholders can be logistically complex and time-consuming.	***Identify trusted and relevant organizations*** Identify organizations already working with young people with cancer (e.g. charities, support groups, and advocacy groups). Use resources like Macmillan’s online directory to find potential partners: https://www.macmillan.org.uk/in-your-area/choose-location.html Host an information session (via webinar, informal meeting or attending partner event) about research with potential partners to discuss collaboration. Learn about the organization’s mission and existing programs or initiatives. Show how the research supports the organization’s existing mission, increasing buy in. Recognize the potential contributions of organizational partners by offering opportunities for co-authorship on publications or co-presentation at conferences. Or offer something back to the communities they work with (e.g. a gathering with food). Provide resources, training, or other forms of support that benefit the partner organization and its people. If available, collaborate with Public Engagement Officers who already have connections with different communities.
**DESIGNING THE STUDY**	
Use of technical language that alienates young people. Materials lacking cultural sensitivity, leading to tokenism or disengagement. Ethical and institutional requirements can feel intimidating or impersonal.	***Simplify communication*** Ensure participant information sheets, consent forms, and study-related communications are clear, jargon-free, and written at an appropriate reading level. Collaborate with young people who have lived experience of cancer (e.g. through an advisory panel, patient advocacy group, or social media) to review information sheets/posters, providing feedback on language, imagery, and overall understanding. For recruitment materials, provide information in multiple formats, such as text, poster, audio, and video, to cater to different learning styles and preferences. Infographics or short explainer videos make complex information more digestible.
Ethical considerations limit how researchers can communicate the benefits of research participation, as researchers must avoid overstating potential advantages and ensure participants have a realistic understanding. Simply including individuals from diverse backgrounds without meaningful involvement can perpetuate tokenism and fail to capture the diversity of their experiences. Materials and messages that are not culturally sensitive can be off-putting or even offensive to members of certain communities.	***Co-create recruitment materials*** Partner with community organizations to co-develop recruitment materials, ensuring they resonate with the target group and ensure cultural appropriateness. Involve young people with lived experience of cancer in the design process, e.g., through advisory boards/patient and public involvement (seek out existing advisory boards if unable to create your own) Ensure that your recruitment materials feature diverse voices and experiences. Highlight the potential benefits in participation information sheets that participants might experience directly, such as the opportunity to connect with others with shared experience, gaining a sense of agency, or contributing to a greater understanding of their experiences.
**OUTREACH & RECRUITMENT**	
Researchers may lack accessible platforms (social media) or opportunities to effectively disseminate research findings to a broader audience, including young people due to time constraints and lack of experience. Platforms such as Instagram and TikTok require a “following” to reach a wider audience. Ethical considerations complicate engagement via digital platforms. Finding young participants willing to share their experiences can be challenging.	***Social media expertise*** Seek out social media training for researchers. Dedicate resources to collaborate with someone with social media expertise/experience who understands the best practices for engaging with young people on platforms like Instagram, TikTok, and YouTube, such as partnering with youth organizations, universities, or freelance professionals. Develop a social media strategy that involves regular, interactive posts, such as polls, stories, and short videos that encourage participation. Involve young people in the design and development of social media content to ensure it is relevant, engaging, and culturally appropriate. Partner with relevant “influencers” or social media personalities who already have a following in the cancer community to help amplify your message. Young PPI members may be able to direct you to who to connect with or have networks themselves. Engage with cancer organizations or relevant through their socials to promote the study.
	***Address privacy concerns (digital content)*** Be mindful of safeguarding and privacy issues. Ensure that all social media content aligns with ethical guidelines and obtain informed consent for any individuals who will be featured in photos, videos, or testimonials. If using video or image content, make sure participants’ faces are blurred, or they are fully aware of how the content will be used, including how long it will be shared, and where it will be posted. Allow participants the option to withdraw consent and remove their content if they choose to.
Finding young people who want to be involved and research and comfortable sharing their experiences can be challenging. Young Champions need training on research methods, communication skills, and ethical considerations and the resources and time to be able to upskill. Maintaining the program’s momentum and retaining Young Champions over time requires ongoing support and recognition. It is important to safeguard the well-being of Young Champions and ensure their involvement is ethical and empowering.	***Partner with Young Champions*** Involve Young Champions to spread the word and engage their peers (see points on collaborations with trusted cancer organizations, healthcare professionals and social media to recruit). Offer training for them to build their research literacy, ethical awareness, and communication skills. Offer flexible participation opportunities, allowing for both in-person and online engagement to accommodate different schedules. Recognize their contributions through certificates, public acknowledgment, or opportunities to co-present at events or conferences. Invite young people to share what they would like to gain from their experiences thus ensuring that participation is meaningful to both researchers and young people and that young people are left feeling valued and empowered in the research process.
Healthcare professionals often have demanding schedules with limited time for activities outside their direct patient care responsibilities. Healthcare professionals may not be aware of all available research studies or how to connect eligible patients with those studies.	***Build relationships with healthcare professionals*** Connect with healthcare providers who work with young people with cancer (e.g., oncologists, social and youth workers). Share one-page summaries of your study, highlighting key details and benefits for patients and health care teams. Make it easy for healthcare professionals to refer eligible patients by providing them with simplified study information, referral forms, and a clear understanding of their role in the recruitment process. Keep healthcare professionals informed throughout the research, with regular updates on study progress and opportunities for them to engage further (e.g. through online meetings, webinars, brief newsletters). Offer CPD or professional development opportunities related to research involvement, ensuring that healthcare professionals feel supported in their role.
	***Easy online sign-up process*** Develop an online registration form that is accessible and straightforward to complete (e.g. QR code sign up). Provide a number or email address for potential participants to ask questions or get more details.
	***Offer incentives for participation*** Provide small incentives (e.g., gift vouchers) for filling out surveys or attending focus groups/interviews. Consult with community partners to ensure incentives are culturally appropriate. Offer travel reimbursements or vouchers to reduce financial barriers for participation (if in person).
**CONDUCTING THE STUDY**	
Power dynamics and institutional processes may create discomfort for participants.	***Create a welcoming environment*** Select locations for in-person sessions that are easily accessible, safe, and welcoming for young people. If possible, use non-clinical settings like community center or University space that feel less intimidating. Offer snacks, drinks, and regular breaks to make the environment more relaxed. If the study requires in-person attendance, allow participants to bring a friend or family member for emotional support if it enables them to feel more comfortable.
	***Foster trust*** Prior to any research activities, offer introductory calls or meetings where participants can ask questions, meet the team, and feel comfortable about what’s involved. Be clear about what is expected of participants (e.g. use a FAQ list) including time commitment and respect their time by ensuring that study sessions are well-organized and do not over run. Foster transparency by providing participants with clear timelines, what they can expect from each stage, and how their data will be used. Ensure that they know their rights, including confidentiality and the ability to withdraw at any time.
**END OF STUDY**	
Researchers often move to other projects due to fixed-term contracts, leaving relationships unmaintained. Demonstrating immediate research impacts is challenging with long project timelines.	***Share findings*** Disseminate findings in accessible formats (e.g., infographics, videos, webinars, podcasts). Share outcomes through social media and newsletters to keep participants and stakeholders informed. Acknowledge participants’ contributions (e.g. certificate of participation, voucher).

Therefore, dedicated funding for Patient and Public Involvement [11,45], social media engagement, and community outreach should be integrated into research budgets. For example, effectively curating social media engagement requires not just financial resources but also dedicated personnel with expertise in online community building. Ultimately, allocating resources to these areas (relationship-building and online engagement), demonstrates a genuine commitment to inclusivity in research.

This study’s strengths include its co-design, participant-centered approach that focuses on the lived experiences of YAs affected by cancer. Diversity in relation to ethnicity, geographic location, and treatment status strengthens the relevance and generalisability of the findings. However, limitations of this study include potential selection bias due to recruitment primarily through cancer support charities, potentially excluding those less connected to such resources, including those with lower health literacy, limited internet access or lower social media engagement. Recruitment of males was challenging, leading to more women than men being represented. In addition, we did not recruit participants at the lower age range, i.e., adolescents of our population of interest and therefore the focus of our research findings have been on the experiences and recommendations of YAs. Additionally, the study’s focus on English-speaking participants may limit its applicability to certain immigrant or minority groups. While the recruitment strategy was intentionally broad in terms of its inclusion, the resulting sample characteristics suggest that findings should be interpreted with caution. This study’s focus on this population should be considered exploratory, with a need to prioritize culturally and linguistically appropriate recruitment strategies and partnerships to ensure broader representation to strengthen generalizability in future research.

**Figure 3.**
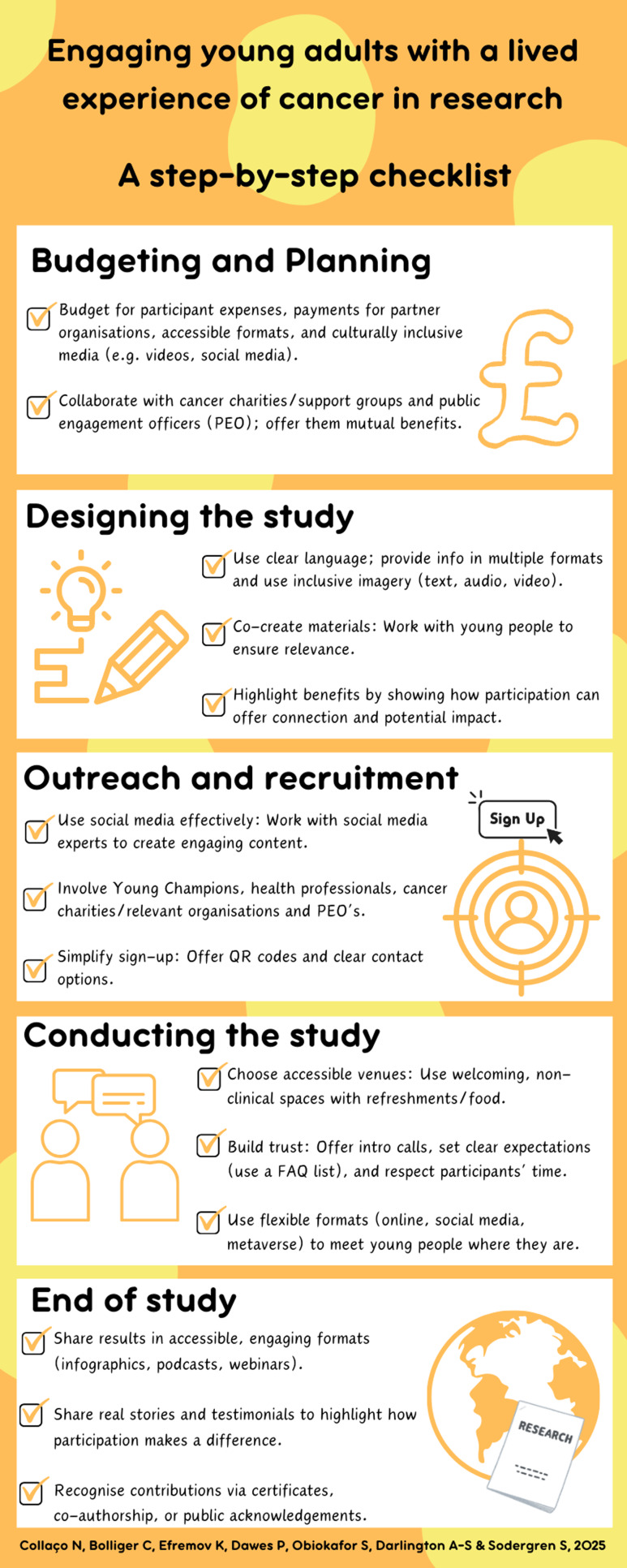
A step by step checklist for researchers on how to engage young adults (YAs) with a lived experience of cancer in research.

This study highlighted key lessons and provides researchers with actionable strategies for overcoming barriers to meaningfully engage YAs in cancer research (see Table 2 and Figure 3). We encourage researchers to apply, evaluate and develop the recommendations further.

## Conclusion

This study provides insights into the barriers and facilitators influencing research participation among YAs with a lived experience of cancer. By adopting the strategies identified here- simplifying language, offering diverse engagement formats, showcasing research impact, building relationships/collaborating with trusted organizations, ensuring dedicated funding for important engagement activities; researchers can create more inclusive and impactful research that genuinely reflects the voices and experiences of YAs. Our research offers practical solutions on how to facilitate the recruitment of YAs to research. Future research should explore the effectiveness of implementing these strategies in diverse research contexts.

## Supporting information

10.1017/cts.2025.10101.sm001 Collaço et al. supplementary material 1 Collaço et al. supplementary material

10.1017/cts.2025.10101.sm002 Collaço et al. supplementary material 2 Collaço et al. supplementary material

## Supplementary material

The supplementary material for this article can be found at https://doi.org/10.1017/cts.2025.10101.

## References

[1] Albritton K , Caligiuri M , Anderson B , et al. Closing the gap: research and care imperatives for adolescents and young adults with cancer: report of the adolescent and young adult oncology progress review group. US DHHS. NIH, NCI, LIVESTRONG Young Adult Alliance, 2006.

[2] Close AG , Dreyzin A , Miller KD , Seynnaeve BK , Rapkin LB. Adolescent and young adult oncology—past, present, and future. CA Cancer J Clin. 2019;69(6):485–496. 31594027 10.3322/caac.21585

[3] Coccia PF. Overview of adolescent and young adult oncology. J Oncol Pract. 2019;15(5):235–237. 31009282 10.1200/JOP.19.00075

[4] Keegan TH , Bleyer A , Rosenberg AS , Li Q , Goldfarb M. Second primary malignant neoplasms and survival in adolescent and young adult cancer survivors. Jama Oncol. 2017;3(11):1554–1557. 28426850 10.1001/jamaoncol.2017.0465 PMC5824209

[5] Bleyer A , Barr R , Hayes-Lattin B , et al. The distinctive biology of cancer in adolescents and young adults. Nat Rev Cancer. 2008;8(4):288–298. 18354417 10.1038/nrc2349

[6] Barr RD , Ferrari A , Ries L , Whelan J , Bleyer WA. Cancer in adolescents and young adults: a narrative review of the current status and a view of the future. Jama Pediatr. 2016;170(5):495–501. 26999630 10.1001/jamapediatrics.2015.4689

[7] Heggie C , Kisby N , Day P , Phillips B. Involving children and young people in supportive cancer care research: the value of FDS grant funding and charitable partnerships. Fac Dent J. 2024;15(4):136–141.

[8] McLaughlin H. Involving young service users as co-researchers: possibilities, benefits and costs. Brit J Soc Work. 2006;36(8):1395–1410.

[9] Wilson O , Daxenberger L , Dieudonne L , et al. A rapid evidence review of young people’s involvement in health research. London: Wellcome; 2020.

[10] L’Hôte M LiSN , Couespel N. Couespel N caught in the middle: Identifying gaps for adolescents and young adults with cancer, 2024.

[11] Taylor RM , Whelan JS , Gibson F , et al. Involving young people in BRIGHTLIGHT from study inception to secondary data analysis: insights from 10 years of user involvement. Res Involv Engagem. 2018;4:1–14. 30607259 10.1186/s40900-018-0135-x PMC6307198

[12] Research NIfH. Inclusive research design to become an NIHR condition of funding. (https://www.nihr.ac.uk/inclusive-research-design-become-nihr-condition-funding) Accessed January 2025.

[13] Cacari-Stone L , Wallerstein N , Garcia AP , Minkler M. The promise of community-based participatory research for health equity: a conceptual model for bridging evidence with policy. Am J Public Health. 2014;104(9):1615–1623. 25033119 10.2105/AJPH.2014.301961 PMC4151933

[14] Anderson AM , Brading L , Swaithes L , et al. Building trust and inclusion with under-served groups: a public involvement project employing a knowledge mobilisation approach. Res Involv Engagem. 2024;10(1):122. 39529198 10.1186/s40900-024-00647-2 PMC11555807

[15] Abrahão R , Alvarez EM , Waters AR , et al. A qualitative study of barriers and facilitators to adolescents and young adults’ participation in cancer clinical trials: Oncologist and patient perspectives. Pediatr Blood Cancer. 2022;69(4):e29479. 34913583 10.1002/pbc.29479

[16] Siembida EJ , Loomans-Kropp HA , Trivedi N , et al. Systematic review of barriers and facilitators to clinical trial enrollment among adolescents and young adults with cancer: identifying opportunities for intervention. Ann Ny Acad Sci. 2020;126(5):949–957. 10.1002/cncr.32675 PMC7029803 31869454

[17] (NIHR) NIfHaCR. National institute for health and care research (NIHR). A practical guide to being inclusive in public involvement in health research: Lessons learnt from the reaching out programme 2021. (https://arc-nenc.nihr.ac.uk/wp-content/uploads/2021/04/NIHR-Reaching-Out_-A-practical-guide-to-being-inclusive-in-public-involvement-in-health-research-Lessons-learnt-from-the-Reaching-Out-programme-April-2021.pdf) Accessed January 2025.

[18] Oveisi N , Cheng V , Taylor D , et al. Meaningful Patient Engagement in Adolescent and Young Adult (AYA) Cancer research: a framework for qualitative studies. Curr Oncol. 2024;31(4):1689–1700. 38668031 10.3390/curroncol31040128 PMC11049004

[19] Tong A , Sainsbury P , Craig J. Consolidated criteria for reporting qualitative research (COREQ): a 32-item checklist for interviews and focus groups. Int J Qual Health C. 2007;19(6):349–357. 10.1093/intqhc/mzm042 17872937

[20] H Finch , Lewis J , Turley C. Focus groups. Qualitative research practice: A guide for social science students and researchers.2003: 2:211–242.

[21] Clarke V , Braun V. Thematic analysis. J Posit Psychol. 2017;12(3):297–298.

[22] Malterud K , Siersma VD , Guassora AD. Sample size in qualitative interview studies: guided by information power. Qual Health Res. 2016;26(13):1753–1760. 26613970 10.1177/1049732315617444

[23] Witham MD , Anderson E , Carroll C , et al. Developing a roadmap to improve trial delivery for under-served groups: results from a UK multi-stakeholder process. Trials. 2020;21:1–9. 32738919 10.1186/s13063-020-04613-7 PMC7395975

[24] Wang RR , Schweitzer JB , Hernandez S , Molina SC , Keegan TH. Strategies for recruitment and retention of adolescent and young adult cancer patients in research studies. J Clin Transl Sci. 2023;7(1):e240. 38028342 10.1017/cts.2023.669 PMC10663769

[25] Roick J , Danker H , Kersting A , et al. Factors associated with non-participation and dropout among cancer patients in a cluster-randomised controlled trial. Eur J Cancer Care. 2018;27(1):e12645. 10.1111/ecc.12645 28134477

[26] Buchanan ND , Block R , Smith AW , Tai E. Psychosocial barriers and facilitators to clinical trial enrollment and adherence for adolescents with cancer. Pediatrics. 2014;133(Supplement_3):S123–S130. 24918211 10.1542/peds.2014-0122I PMC4258829

[27] Fern LA , Taylor RM. Enhancing accrual to clinical trials of adolescents and young adults with cancer. Pediatr Blood Cancer. 2018;65(9):e27233. 29749691 10.1002/pbc.27233

[28] Mitchell S , Bragg A , Moldovan I , et al. Stigma as a barrier to participant recruitment of minority populations in diabetes research: development of a community-centered recruitment approach. JMIR Diabetes. 2021;6(2):e26965. 33938811 10.2196/26965 PMC8129881

[29] Gafari O , Bahrami-Hessari M , Norton J , et al. Building trust and increasing inclusion in public health research: co-produced strategies for engaging UK ethnic minority communities in research. Public Health. 2024;233:90–99. 38865828 10.1016/j.puhe.2024.05.007

[30] Whittaker L , Dean JA , Veiga C , et al. Radiation reveal: moving from research engagement to involvement. Brit J Cancer. 2024;130(10):1593–1598. 38615107 10.1038/s41416-024-02648-0 PMC11091114

[31] Gorman JR , Roberts SC , Dominick SA , Malcarne VL , Dietz AC , Su HI. A diversified recruitment approach incorporating social media leads to research participation among young adult-aged female cancer survivors. J Adolesc Young Adul. 2014;3(2):59–65. 10.1089/jayao.2013.0031 PMC4048966 24940529

[32] NLiS ML’Hôte , Couespel N. Caught in the middle: Identifying gaps for Adolescents and Young Adults (AYAs) with cancer 2024;).

[33] Taylor RM , Solanki A , Aslam N , Whelan JS , Fern LA. A participatory study of teenagers and young adults views on access and participation in cancer research. Eur J Oncol Nurs. 2016;20:156–164. 26251363 10.1016/j.ejon.2015.07.007

[34] Ford M , Wahlquist A , Blake R , et al. Assessing an intervention to improve clinical trial perceptions among predominately African-american communities in south Carolina. Progress in Community Health Partnerships: Research, Education, and Action. 2012;6(3):249–263. 22982839 10.1353/cpr.2012.0038 PMC4180673

[35] Mitschke DB , Cassel K , Higuchi P. Empowering natural clinical trial advocates: nurses and outreach workers. Pac Health Dialog. 2007;14(1):135–141. 19772149

[36] Rafie C , Ayers A , Cadet D , Quillin J , Hackney MH. Reaching hard to reach populations with hard to communicate messages: efficacy of a breast health research champion training program. J Cancer Educ. 2015;30:599–606. 25171905 10.1007/s13187-014-0720-0 PMC4345135

[37] Oduola S , Wykes T , Robotham D , Craig TK. What is the impact of research champions on integrating research in mental health clinical practice? A quasiexperimental study in South London, UK. BMJ Open. 2017;7(9):e016107. 10.1136/bmjopen-2017-016107 PMC5595181 28899890

[38] Prinjha S , Miah N , Ali E , Farmer A. Including seldom heardviews in research: opportunities, challenges and recommendations from focus groups with british south Asian people with type 2 diabetes. Bmc Med Res Methodol. 2020;20:1–11. 10.1186/s12874-020-01045-4 PMC7296709 32539718

[39] Preston J , Lappin E , Ainsworth J , Wood CL , Dimitri P. Involving children and young people as active partners in paediatric health research. Paediatr Child Health. 2024;34(1):11–16.

[40] Monge-Montero C , O’Callaghan S , Rizvi K , Košir U , Gîrbu V. Recommendations for equitable, diverse, and inclusive cancer care in Europe 2024. (https://efaidnbmnnnibpcajpcglclefindmkaj/https://beatcancer.eu/wp-content/uploads/2024/04/YCE_Recommendations_for_Equitable_Diverse_and_Inclusive_Cancer_Care_in_Europe.pdf) Accessed January 2025.

[41] Equality NIHR. Diversity and inclusion toolkit, (https://www.rssleicesterresources.org.uk/edi-toolkit) Accessed October 2024.

[42] Trust TC. Equity, diversity, inclusion strategy 2022-2024 2022. (https://efaidnbmnnnibpcajpcglclefindmkaj/https://www.teenagecancertrust.org/sites/default/files/2022-04/EDI-strategy-2022.pdf) Accessed January 2025.

[43] C.R. UK. Cancer research UK’s equality, diversity and inclusion strategy 21-23 2021. (https://efaidnbmnnnibpcajpcglclefindmkaj/https://www.cancerresearchuk.org/sites/default/files/cancer_research_uk_equality_diversity_and_inclusion_strategy_2021_2023.pdf) Accessed January 2025.

[44] Fern LA , Lewandowski JA , Coxon KM , Whelan J. Available, accessible, aware, appropriate, and acceptable: a strategy to improve participation of teenagers and young adults in cancer trials. Lancet Oncol. 2014;15(8):e341–e350. 24988937 10.1016/S1470-2045(14)70113-5

[45] Teodorowski P. Involvement and Engagement of Seldom-Heard Communities in Big Data Research (Doctoral dissertation). The University of Liverpool (United Kingdom); 2023.

