# Supplementary material for: “Meet us where we’re at:” Towards engaging and inclusive research with young adults with a lived experience of cancer
Source: J Clin Transl Sci. 2025 Jul 16;9(1):e159. doi: 10.1017/cts.2025.10101 (PMC12392343; doi:10.1017/cts.2025.10101)
Supplement: Collaço et al. supplementary material 2 — Collaço et al. supplementary material [file S2059866125101015sup002.docx]

Discussion Topics

1. **Identifying facilitators and barriers**
2. You have shared some ideas / proposals for how to engage / encourage young people across different groups to be involved as research partners / join an advisory group. Can you think of anything additional we could do (also think about engaging with the broader group of young people with cancer)?
3. What do you think are the challenges (in general and specific to under-represented groups) to engaging with young people and sustaining involvement over time?
4. **How to adopt an inclusive approach**

People who we don’t usually hear from you- education of healthcare professionals as gatekeepers

What does diverse background mean to you (prompt: personally and professionally)? If we were to develop such a group, are there certain people you think should definitely be involved / invited?

It could be argued that (or you have mentioned that) we need to have a good representation across young people. How do we go about achieving this?
